# Multitarget antitumor effects of panacis japonici rhizoma

**DOI:** 10.3389/fphar.2025.1591638

**Published:** 2025-12-03

**Authors:** Xixing Fang, Xiao Yang, Changyuan Zhou, Di Zeng, Zehua Hu, Xing Tu, Bao Yang, Yuanxin Hou

**Affiliations:** 1 Hubei Provincial Key Laboratory of Occurrence and Intervention of Rheumatic Diseases, Hubei Minzu University, Enshi, China; 2 Medical School, Hubei Minzu University, Enshi, China; 3 Hubei Provincial Clinical Medical Research Center for Nephropathy, Hubei Minzu University, Enshi, China; 4 Minda Hospital of Hubei Minzu University, Enshi, China

**Keywords:** Panacis Japonici Rhizoma, zhujieshen, tumors, total saponins, chikusetsusaponin IVa, chikusetsusaponin IV, chikusetsusaponin V

## Abstract

*Panax japonicus* C.A. Mey., belonging to the genus Panax in the Araliaceae family, is a perennial drug plant. Its rhizomes, known traditional Chinese medicine (TCM) as Zhujieshen (Panacis Japonici Rhizoma), have a long history spanning thousands of years. This review systematically summarizes the multitarget antitumor effects of Zhujieshen and its bioactive metabolites, such as total saponins (TSPJ) and specific chikusetsusaponins (IVa, IV, V). Preclinical studies demonstrate broad anticancer activities against lung, liver, cervical, ovarian, prostate, and colorectal cancers by promoting apoptosis, suppressing proliferation, inhibiting metastasis, and enhancing chemosensitivity. Pharmacological investigations reveal that these effects are mediated through modulation of key signaling pathways, including PI3K/Akt, PKCα-ERK1/2, TLR4/NF-κB, and Wnt/β-catenin, which act on molecular targets like MMP-2, MMP-9, and Caspase-3. This collective regulation reduces inflammatory cytokine secretion, curtails tumor growth and spread, and increases sensitivity to chemotherapy.

## 1 Introduction

**FIGURE 1:**
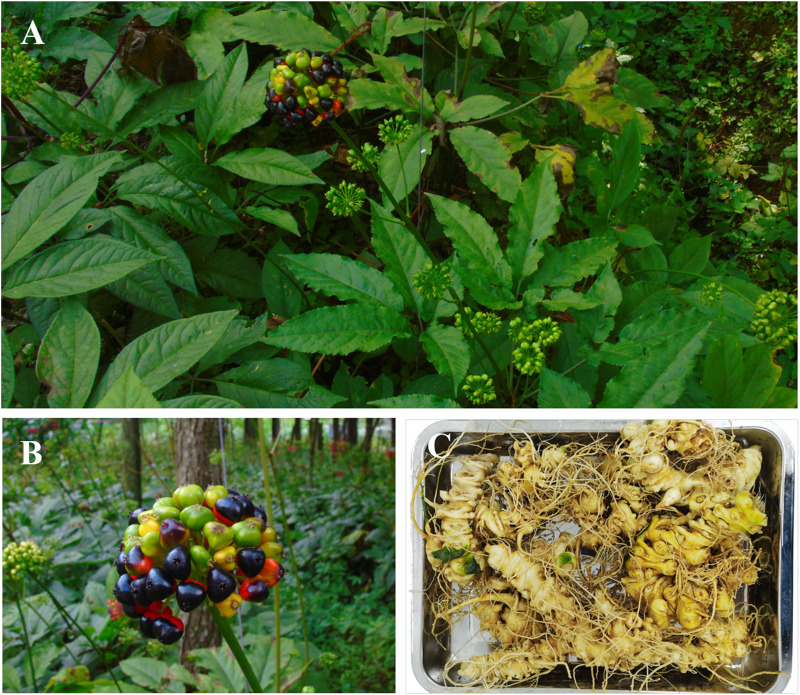
Whole plants **(A)**, fresh firuits **(B)**, fresh rhizomes **(C)** of *Panax japonicus*.

Belonging to the Araliaceae family and classified under the genus *Panax*, *Panax japonicus* C.A. Mey. is a perennial drug plant (Yang et al., 2014). Its geographical distribution covers humid and semi-humid areas southward of China’s Yellow River basin, extending to Vietnam, Nepal, Myanmar, Japan, and North Korea (Morita et al., 1985; Zhang et al., 2015a). Zhujieshen, named for its elongated rhizomes with a horizontal growth pattern resembling bamboo joints, has been prominently employed in Chinese traditional medicine for thousands of years (Figure 1). Zhujieshen was initially documented in the ancient medical text *Bencao Yuanshi* (1612) from the Ming Dynasty and has subsequently been included in the Pharmacopoeia of the People’s Republic of China since 1977. In Tujia medicine, Zhujieshen is classified as a classic “Qi” medicine. It is used to strengthen the body, relieve cough, eliminate phlegm, disperse blood stasis, stop bleeding, reduce swelling, and alleviate pain. It is commonly applied to treat cough with hemoptysis due to tuberculosis, traumatic injuries, excessive cough with phlegm, and weakness following illness (Zhang et al., 2015b). Previous pharmacological research has demonstrated that extracts and active metabolites from Zhujieshen exhibit significant pharmacological activities, including antitumor, anti-fatigue, immunomodulatory, cardioprotective, sedative, analgesic, and anti-rheumatoid arthritis effects (Jie et al., 2015).

In 2022, the estimated worldwide incidence of cancer reached nearly 20 million cases, resulting in approximately 9.7 million fatalities related to cancer (Kaur et al., 2023). The *Global Cancer Statistics 2022* report, published by CA: A Cancer Journal for Clinicians on 4 April 2024, was collaboratively produced by the International Agency for Research on Cancer (IARC) and the American Cancer Society (ACS) (Bray et al., 2024). This extensive publication presents detailed mortality and incidence analyses covering 36 cancer varieties across 185 nations. According to this report, the total global cancer incidence in 2022 was about 19.96 million, of which China accounted for 24.1%, ranking first globally. Regarding cancer-related mortality, global deaths reached approximately 9.7 million, with China accounting for 26.5%, also ranking first (Santucci et al., 2020). Despite this severe global health challenge, no definitive clinical cure exists for malignant tumors. Conventional treatment strategies primarily include surgical therapy, pharmacotherapy, radiotherapy, targeted therapy, and immunotherapy. Surgical therapy and radiotherapy primarily relieve symptoms but pose high risks, cause substantial trauma, carry significant recurrence rates, and induce numerous adverse reactions, often resulting in severe damage to tissues, organs, and immune function. Consequently, pharmacotherapy plays a central role in the comprehensive management of malignant tumors. However, drug toxicity and resistance significantly limit the efficacy of first-line antitumor agents. There is an urgent clinical demand for anticancer drugs that provide targeted treatment with minimal side effects while improving overall body functions (Suazo-Zepeda et al., 2021).

In recent years, the Western medical community has increasingly recognized the therapeutic potential of TCM extracts and bioactive metabolites in combating malignant tumors. Notable examples include camptothecin, paclitaxel, curcumol, and vincristine. TCM exhibits distinctive therapeutic advantages in oncology management through multi-metabolite integration and multi-pathway synergistic mechanisms. This systemic pharmacological approach allows comprehensive modulation of various tumorigenic targets and signaling cascades, reflecting the holistic therapeutic philosophy of TCM. Furthermore, compared with Western medical therapies, TCM provides additional benefits, such as replenishing qi and generating blood, harmonizing the zang-fu organs, enhancing immunity, addressing both deficiency and excess conditions, and causing fewer adverse reactions. TCM not only alleviates tumor symptoms but also improves patients’ quality of life and prolongs survival. Therefore, developing potent anticancer agents from TCM represents a promising therapeutic strategy.

This review systematically examines the antitumor mechanisms of total saponins and bioactive metabolites derived from Panacis Japonici Rhizoma (Zhujieshen) (Chen H. M. et al., 2023). It aims to elucidate their specific therapeutic targets in oncology, providing valuable references for fundamental research and clinical application of Zhujieshen in tumor treatment. Additionally, this review offers new insights and a foundation for the development and utilization of medicinal plant resources within this genus.

## 2 Materials and methods

### 2.1 Search strategy

The preparation of this review followed the Preferred Reporting Items for Systematic Evaluation and Meta-Analysis (PRISMA) guidelines (Page et al., 2022), and the literature was retrieved in PubMed (https://pubmed.ncbi), Web of Science (https://www.webofscience.com), and CNKI (http://www.cnki.net) databases as of March 2025, with the main keywords of “Panacis japonici rhizoma,” “*Panax japonicus*,” “Zhujieshen,” “antitumor,” “anticancer,” “total saponins,” and “Chikusetsusaponin”.

### 2.2 Eligibility and exclusion criteria

All the studies related to the keywords were considered and subsequently screened according to the following eligibility criteria: 1) articles written in English or Chinese; 2) most of the experimental studies on Panacis Japonici Rhizoma have focused on its botany, phytochemistry, and pharmacology; 3) the focus of the study was on evaluating the antitumor effects of extracts or isolated compounds from Panacis Japonici Rhizoma.

**FIGURE 2:**
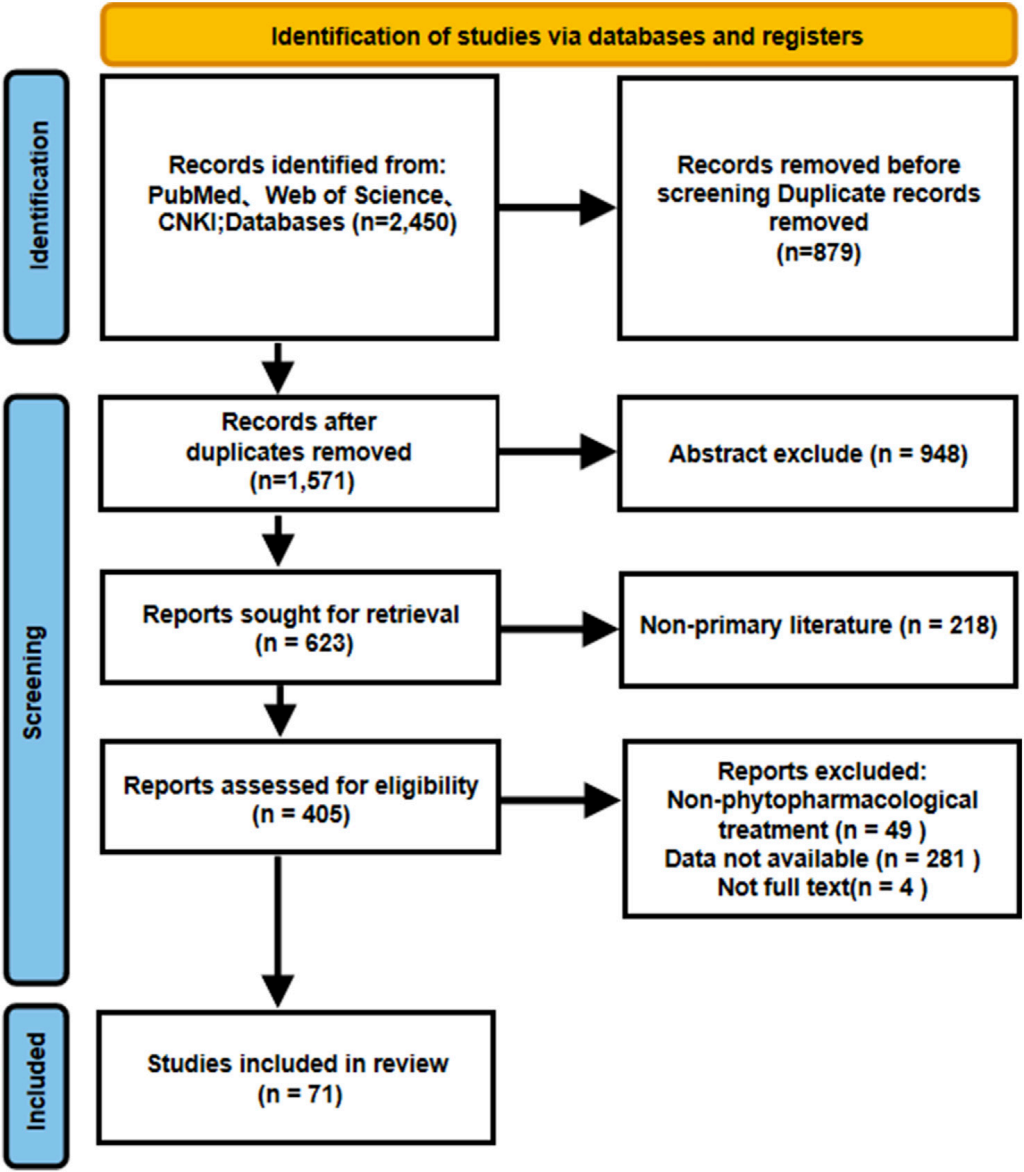
Literature screening process.

The exclusion criteria included: 1) studies involving human subjects or combined herbal formulations without isolated evaluation of Zhujieshen; 2) publications in the category of reviews, editorials, conference abstracts, or other non-primary literature; 3) duplicate publications or studies with insufficient methodological detail. Relevant eligibility and exclusion criteria are briefly described in the PRISMA flowchart (Figure 2).

## 3 Phytochemistry

**FIGURE 3:**
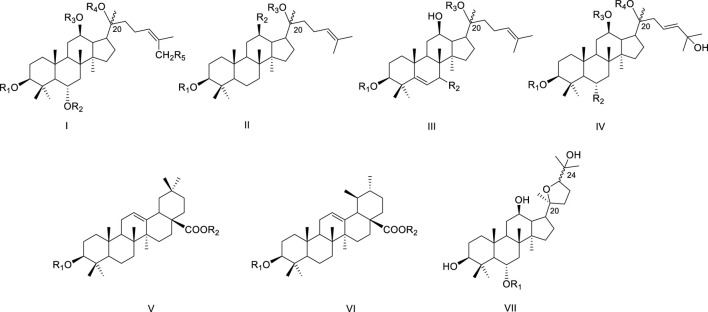
The skeleton of triterpenoids isolated from the Zhujieshen.

Zhujieshen contains abundant saponins, with 86 distinct triterpenoid saponins isolated and structurally elucidated to date (Figure 3; Table 1). These triterpenoid saponins are glycosidic metabolites composed of triterpenoid aglycones bound to one or multiple sugar residues. Based on their aglycone structure, they can be divided into dammarane-type tetracyclic and oleanane-type pentacyclic triterpenoids. The dammarane-type category encompasses subtypes such as protopanaxadiol, protopanaxatriol, and ocotillol, whereas oleanane-type saponins include those derived from oleanolic acid and ursolic acid. Triterpenoid saponins, including CSIVa (46), CSIV (47), and CSV (48) are abundant in Zhujieshen, as quantified in previous studies. As stipulated by the Chinese Pharmacopoeia (2025 edition), the concentrations of CSIVa and CSV in Zhujieshen must individually meet or exceed 1.5%. Our previous studies showed that the content of CSIVa, CSIV, and CSV in different batches of Zhujieshen ranged from 0.2% to 3.1%, 1.9%–6.1%, and 4.4%–9.5%, respectively. Correspondingly, their proportions within total saponins were 5.2%, 23.8%, and 36.3% (Long et al., 2022).

**TABLE 1:** Information on triterpenoids isolated from Zhujieshen.

No	Compound name	Skeleton	R_1_	R_2_	R_3_	R_4_	R_5_	C_20_	C_24_	Ref.
1	Ginsenoside Re	Ⅰ	H	Glc (2←1)Rha	H	Glc	H	*S*	-	Morita et al. (1983), Morita et al. (1985), Yoshizaki et al. (2013), Zhou et al. (2011), Zou et al. (2002b)
2	Ginsenoside Rg_1_	Ⅰ	H	Glc	H	Glc	H	*S*	-	Chan et al. (2011), Morita et al. (1983), Morita et al. (1985), Yoshizaki et al. (2013), Zhou et al. (2011), Zou et al. (2002b)
3	Ginsenoside Rg_2_	Ⅰ	H	Glc (2←1)Rha	H	H	H	*S*	-	Morita et al. (1983), Morita et al. (1985), Yoshizaki et al. (2013)
4	(20*S*)-Ginsenoside Rh_1_	Ⅰ	H	Glc	H	H	H	*S*	-	Yoshizaki et al. (2013), Zhou et al. (2011)
5	(20*R*)-Ginsenoside Rh_1_	Ⅰ	H	Glc	H	H	H	*R*	-	Liu et al. (2016a)
6	Notoginsenoside R_1_	Ⅰ	H	Glc (2←1)Xyl	H	Glc	H	*S*	-	Morita et al. (1985), Yoshizaki et al. (2013), Zhou et al. (2011), Zou et al. (2002b)
7	Notoginsenoside R_2_	Ⅰ	H	Glc (2←1)Xyl	H	H	H	*S*	-	Morita et al. (1983), Morita et al. (1985), Yoshizaki et al. (2013), Zhou et al. (2011)
8	Yesanchinosides R_1_	Ⅰ	H	Glc	H	H	OH	*S*	-	Zhou et al. (2011)
9	Yesanchinosides R_2_	Ⅰ	H	Glc (2←1)Xyl	H	H	OH	*S*	-	Zhou et al. (2011)
10	6‴-*O*-acetylginsenoside Re	Ⅰ	H	Glc (2←1)Rha	H	Glc-(6-*O*-AC)	H	*S*	-	Zhou et al. (2011)
11	Ginsenoside Rf	Ⅰ	H	Glc (2←1)Glc	H	H	H	*S*	-	Chan et al. (2011), Zhou et al. (2011)
12	Chikusetsusaponin IVc	Ⅰ	H	Glc (2←1)Rha	H	Glc	H	*S*	-	Lin and Shoji (1979)
13	(20*S*)-Protopanaxatriol	Ⅰ	H	H	H	H	H	*S*	-	Morita et al. (1985)
14	Yesanchinoside D	Ⅰ	H	Glc (6-*O*-AC)	H	Glc	H	*S*	-	Zou et al. (2002b)
15	Yesanchinoside E	Ⅰ	H	Glc (2←1)Rha	H	Glc (6←1)Glc	H	*S*	-	Zou et al. (2002b)
16	Yesanchinoside F	Ⅰ	H	Glc-[(2←1)Rha]- 6-*O*-AC	H	Glc (6←1)Glc	H	*S*	-	Zou et al. (2002b)
17	Notoginsenoside R6	Ⅰ	H	Glc	H	Glc (6←1)Glc*	H	*S*	-	Zou et al. (2002b)
18	20-*O*-Glu-ginsenoside Rf	Ⅰ	H	Glc (2←1)Glc	H	Glc	H	*S*	-	Zou et al. (2002b)
19	Panajaponol A	Ⅰ	H	Glc (2←1)Glc	H	H	OH	*S*	-	Chan et al. (2011)
20	(20*S*)-Protopanaxatriol	ⅠⅠ	H	OH	H	-	-	*S*	-	Morita et al. (1983), Morita et al. (1985), Yoshizaki et al. (2013)
21	Ginsenoside F_2_	ⅠⅠ	Glc	OH	Glc	-	-	*S*	-	Yoshizaki et al. (2013)
22	Chikusetsusaponin Ia	ⅠⅠ	Glc (6←1)Xyl	OH	H	-	-	*S*	-	Morita et al. (1985), Yoshizaki et al. (2013)
23	Chikusetsusaponin VII	ⅠⅠ	Glc (6←1)Xyl	OH	Glc (6←1)Glc	-	-	*S*	-	Yoshizaki et al. (2013)
24	Ginsenoside Rb_1_	ⅠⅠ	Glc (2←1)Glc	OH	Glc (6←1)Glc		-	*S*	-	Chan et al. (2011), Morita et al. (1985), Yoshizaki et al. (2013), Zhou et al. (2011), Zou et al. (2002a)
25	Ginsenoside Rb_3_	ⅠⅠ	Glc (2←1)Glc	OH	Glc (6←1)Xyl		-	*S*	-	Yoshizaki et al. (2013), Zou et al. (2002a)
26	Ginsenoside Rc	ⅠⅠ	Glc (2←1)Glc	OH	Glc (6←1)Ara		-	*S*	-	Morita et al. (1985), Yoshizaki et al. (2013), Zou et al. (2002a)
27	Ginsenoside Rd	ⅠⅠ	Glc (2←1)Glc	OH	Glc		-	*S*	-	Chan et al. (2011), Morita et al. (1983), Yoshizaki et al. (2013), Zhou et al. (2011), Zou et al. (2002a)
28	Notoginsenoside Fe	ⅠI	Glc	OH	Glc (6←1)Ara	-	-	*S*	-	Yoshizaki et al. (2013), Zou et al. (2002a)
29	Chikusetsusaponin III	ⅠⅠ	Glc (6←1)-[(2←1)Glc]-Xyl	OH	H	-	-	*S*	-	Kondo et al. (1970), Lin et al. (1976), Morita et al. (1985), Yoshizaki et al. (2013)
30	Chikusetsusaponin VI	ⅠⅠ	Glc (6←1)-[(2←1)Glc]-Xyl	OH	Glc (6←1)Glc	-	-	*S*	-	Yoshizaki et al. (2013)
31	Chikusetsusaponin FK_6_	ⅠⅠ	Glc (6←1)-[(2←1)Glc]-Xyl	OH	Glc	-	-	*S*	-	Yoshizaki et al. (2013)
32	Gypenoside XVII	ⅠⅠ	Glc	OH	Glc (6←1)Glc	-	-	*S*	-	Yoshizaki et al. (2013), Zhou et al. (2011)
33	Yesanchinoside J	ⅠⅠ	Glc-[(2←1)Glc]- 6-*O*-AC	OH	Glc (6←1)Glc (6←1)Xyl	-	-	*S*	-	Zou et al. (2002a)
34	Notoginsenoside R_4_	ⅠⅠ	Glc (2←1)Glc	OH	Glc (6←1)Glc (6←1)Xyl	-	-	*S*	-	Zou et al. (2002a)
35	Notoginsenoside Fa	ⅠⅠ	Glc (2←1)Glc (2←1)Xyl	OH	Glc (6←1)Glc	-	-	*S*	-	Zou et al. (2002a)
36	Notoginsenoside Fc	ⅠⅠ	Glc (2←1)Glc (2←1)Xyl	OH	Glc (6←1)Glc	-	-	*S*	-	Zou et al. (2002a)
37	Yesanchinoside I	ⅠⅠ	Glc (2←1)Glc	H	Glc (6←1)Glc (6←1)Xyl	-	-	*S*	-	Zou et al. (2002a)
38	(20*S*)-Ginsenoside Rg_3_	ⅠⅠ	Glc (2←1)Glc	H	H	H	H	*S*	-	
39	(20*R*)-Ginsenoside Rg_3_	ⅠⅠ	Glc (2←1)Glc	H	H	H	H	*R*	-	
40	Yesanchinoside G	III	Glc (2←1)Glc	OH	Glc (6←1)Xyl	-	-	*S*	-	Zou et al. (2002a)
41	Notoginsenoside G	III	Glc (2←1)Glc	OH	Glc	-	-	*S*	-	Zou et al. (2002a)
42	Quinquenoside IV	III	Glc (2←1)Glc	OH	Glc (6←1)Glc	-	-	*S*	-	Zou et al. (2002a)
43	Yesanchinoside H	IV	Glc (2←1)Glc	H	H	Glc (6←1)Xyl	-	*S*	-	Zou et al. (2002a)
44	Vinaginsenoside R_15_	IV	H	OGlc	H	Glc	-	*S*	-	Zhou et al. (2011)
45	Oleanolic acid 28-*O*-β-D-glucopyranoside	V	H	Glc	-	-	-	-	-	Chan et al. (2011), Liu et al. (2016a), Zhou et al. (2011), Cai et al. (1982)
46	Chikusetsusaponin IVa	V	GlcA	Glc	-	-	-	-	-	Chan et al. (2011), Liu et al. (2016a), Morita et al. (1983), Morita et al. (1985), Yoshizaki et al. (2013), Zhou et al. (2011), Zou et al. (2012)
47	Chikusetsusaponin IV	V	GlcA (4←1)Ara	Glc	-	-	-	-	-	Liu et al. (2016a), Morita et al. (1983), Morita et al. (1985), Yoshizaki et al. (2013), Zhou et al. (2011), Cai and Xiao (1984), Zou et al. (2012)
48	Chikusetsusaponin V	V	GlcA (2←1)Glc	Glc	-	-	-	-	-	Chan et al. (2011), Kondo et al. (1971), Liu et al. (2016a), Morita et al. (1983), Morita et al. (1985), Yoshizaki et al. (2013), Zhou et al. (2011), Zou et al. (2012)
49	28-Desglucosylchikusetsusaponin IV	V	GlcA (4←1)Ara	H	-	-	-	-	-	Yoshizaki et al. (2013), Zhou et al. (2011)
50	Zingibroside R_1_	V	GlcA (2←1)Glc	H	-	-	-	-	-	Yoshizaki et al. (2013)
51	Oleanolic acid 3-*O*-β-D-(6′-methyl ester)glucuronopyranoside	V	GlcA methyl ester	H	-	-	-	-	-	Zhou et al. (2011)
52	Chikusetsusaponin IVa methyl ester	V	GlcA methyl ester	Glc	-	-	-	-	-	Chan et al. (2011), Liu et al. (2016a), Zhou et al. (2011)
53	Oleanolic acid 3-*O*-β-D-glucopyranosyl-(1→2)- β-D-(6′-methyl ester)glucuronopyranoside	V	GlcA methyl ester (2←1)Glc	H	-	-	-	-	-	Zhou et al. (2011)
54	Chikusetsusaponin V methyl ester	V	GlcA methyl ester (2←1)Glc	Glc	-	-	-	-	-	Liu et al. (2016a), Zhou et al. (2011)
55	Chikusetsusaponin IV methyl ester	V	GlcA methyl ester (4←1)Ara	Glc	-	-	-	-	-	Chan et al. (2011), Liu et al. (2016a), Zhou et al. (2011)
56	Polysciassaponin P5	V	GlcA (2←1)Glc	H	-	-	-	-	-	Zhou et al. (2011)
57	Baisanqisaponin A	V	GlcA (6←10‴’)X	Glc	-	-	-	-	-	Liu et al. (2016a)
58	Baisanqisaponin B	V	GlcA (6←10‴’)-[(4←1)Ara]X	Glc	-	-	-	-	-	Liu et al. (2016a)
59	Baisanqisaponin C	V	GlcA (6←10‴’)-[(2←1)Glc]X	H	-	-	-	-	-	Liu et al. (2016a)
60	Chikusetsusaponin V ethyl ester	V	GlcA ethyl ester (2←1)Glc	H	-	-	-	-	-	Liu et al. (2016a)
61	Chikusetsusaponin IVa ethyl ester	V	GlcA ethyl ester	Glc	-	-	-	-	-	Liu et al. (2016a)
62	Chikusetsusaponin IVa butyl ester	V	GlcA butyl ester	Glc	-	-	-	-	-	Chan et al. (2011), Liu et al. (2016a)
63	Taibaienoside I	V	GlcA butyl ester (4←1)Ara	Glc	-	-	-	-	-	Liu et al. (2016a)
64	Taibaienoside II	V	GlcA ethyl ester (4←1)Ara	Glc	-	-	-	-	-	Liu et al. (2016a)
65	28-Desglucosylchikusetsusaponin IVa butyl ester	V	GlcA butyl ester	H	-	-	-	-	-	Liu et al. (2016a)
66	Chikusetsusaponin V butyl ester	V	GlcA butyl ester (2←1)Glc	Glc	-	-	-	-	-	Liu et al. (2016a)
67	Pseudoginsenoside RT_1_ butyl ester	V	GlcA butyl ester (2←1)Xyl	Glc	-	-	-	-	-	Chan et al. (2011), Liu et al. (2016a)
68	Chikusetsusaponin II	V	Glc (6←1)Glc	H	-	-	-	-	-	Lin and Shoji (1979)
69	Chikusetsusaponin Ib	V	GlcA (6←1)-[(4←1)Ara]-Glc	H	-	-	-	-	-	Lin et al. (1976), Morita et al. (1985)
70	Hemsgiganoside B	V	GlcA	Glc (6←1)Glc	-	-	-	-	-	Zou et al. (2012)
71	Oleanolic acid	V	H	H	-	-	-	-	-	Chan et al. (2011), Morita et al. (1985)
72	Taibaienoside I	V	GlcA butyl ester (4←1) Ara	Glc	-	-	-	-	-	Chan et al. (2011)
73	Stipuleanoside R_2_	V	GlcA (4←1)-[(3←1)Glc]- Ara	Glc	-	-	-	-	-	Chan et al. (2011)
74	Pseudoginsenoside RT_1_	V	GlcA (2←1)Xyl	Glc	-	-	-	-	-	Chan et al. (2011)
75	Pseudoginsenoside RT_1_ methyl ester	V	GlcA methyl ester (2←1)Xyl	Glc	-	-	-	-	-	Chan et al. (2011)
76	Cynarasaponin C	VI	GlcA	Glc	-	-	-	-	-	Zou et al. (2012)
77	Majonoside R_1_	VII	Glc (2←1)Glc	-	-	-	-	*S*	*S*	Chan et al. (2011), Morita et al. (1983)
78	Majonoside R_2_	VII	Glc (2←1)Xyl	-	-	-	-	*S*	*S*	Morita et al. (1983)
79	Pseudo-ginsenoside F_11_	VII	Glc (2←1)Rha		-	-		*S*	*R*	Morita et al. (1983), Zou et al. (2002b)
80	Yesanchinoside A	VII	Glc-[(2←1)Glc]- 6-*O*-AC	-	-	-	-	*S*	*S*	Zou et al. (2002b)
81	Yesanchinoside B	VII	Glc (6←1)-[(2←1)Glc]-Glc*	-	-	-	-	*S*	*S*	Zou et al. (2002b)
82	Yesanchinoside C	VII	Glc (2←1)Glc (2←1)Xyl	-	-	-	-	*S*	*S*	Zou et al. (2002b)
83	Vina-ginsenoside R_1_	VII	Glc-[(2←1)Rha]- 6-*O*-AC	-	-	-	-	*S*	*S*	Zou et al. (2002b)
84	Pseudoginsenoside RT_4_	VII	Glc	-	-	-	-	*S*	*S*	Zou et al. (2002b)
85	Vina-ginsenoside R_2_	VII	Glc-[(2←1)Xyl]- 6-*O*-AC	-	-	-	-	*S*	*S*	Zou et al. (2002b)
86	Vina-ginsenoside R_6_	VII	Glc (6←1)-[(2←1) Xyl]-Glc	-	-	-	-	*S*	*S*	Zou et al. (2002b)

Glc: β-D-glucopyranosyl; Glc*: α-D-glucopyranosyl; GlcA: β-D-glucuronopyranosyl; Xyl: β-D-xylopyranosyl; Ara: α-L-arabinofuranosyl; Rha: α-L-rhamnopyranosyl; AC: acetyl; X: polyacetylenic.

## 4 Pharmacological activities

**FIGURE 4:**
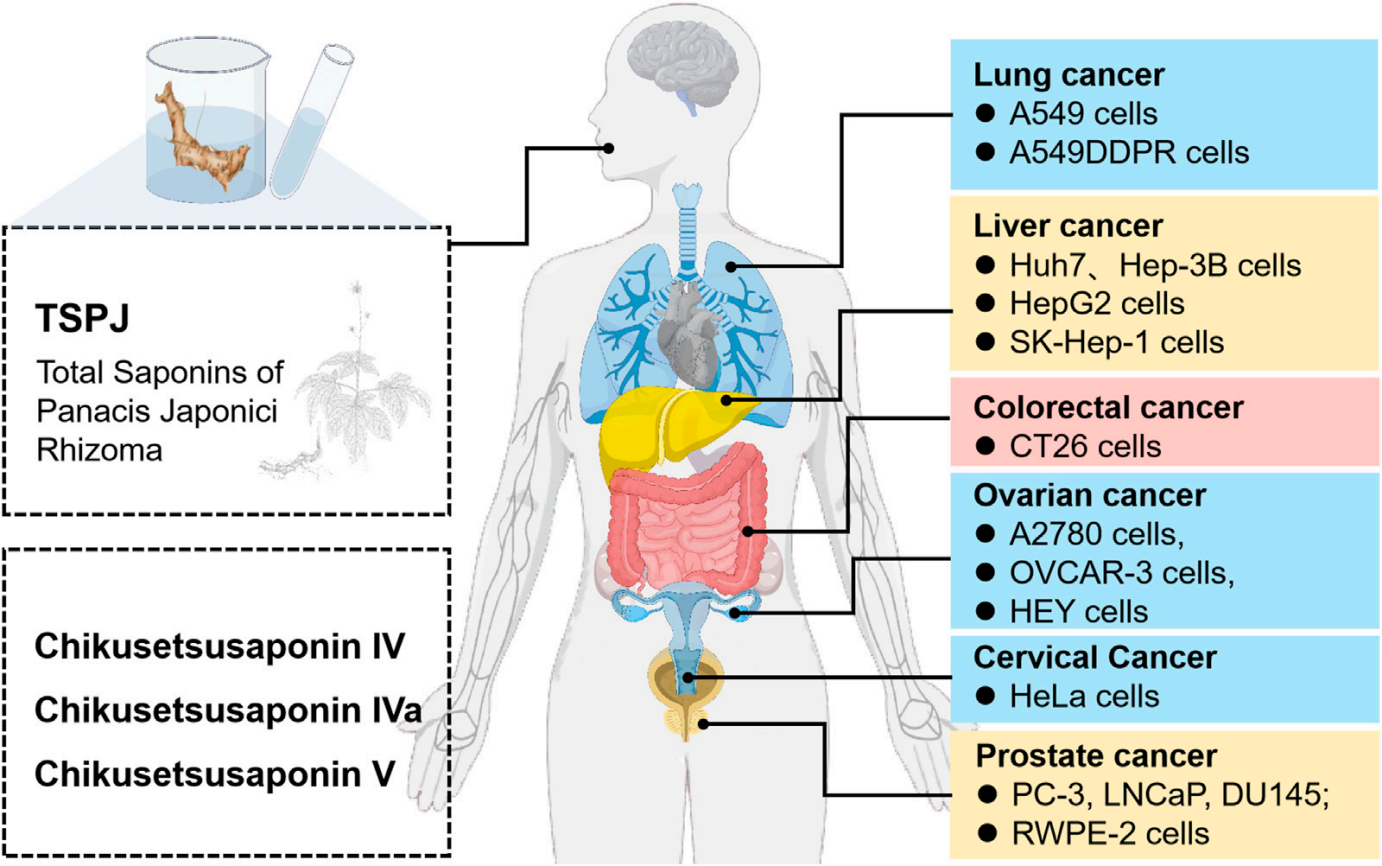
Antitumor models of Zhujieshen.

Extracts derived from Zhujieshen and their active metabolites have been reported to exhibit anticancer activities against various malignancies, such as lung, prostate, and colon cancers; however, the evidence is primarily derived from preclinical studies, and clinical relevance remains to be validated. The underlying mechanisms by which these extracts exert therapeutic effects encompass the suppression of tumor cell growth, promotion of apoptotic cell death, reduction of tumor migration and invasion, potentiation of chemotherapeutic sensitivity, regulation of immune functions, and enhancement of immune cell-mediated recognition and elimination of tumor cells. Additionally, these metabolites ameliorate cachexia in cancer patients (Figure 4). Due to the multimetabolite and multitarget characteristics of Zhujieshen, the antitumor mechanisms of its bioactive metabolites may exhibit specificity toward different tumor types (Table 2).

**TABLE 2:** Anti-tumor activities of Zhujieshen.

Pathology	Metabolites	Cell lines or models	Doses	Duration	Result	Remarks	References
Lung cancer	TSPJ	*In vitro*: A549 human lung adenocarcinoma cells Controls: Vehicle (0.1% DMSO); positive (cisplatin, 10 μM)	0–100 μg/mL (IC_50_ = 50 μg/mL)	48 h	↑ Caspase-3 activation; ↓ Bcl-2/Bax ratio; inhibition of TLR4/NF-κB signaling; ↓ IL-6 and TNF-α secretion	Significant discrepancy between *in vitro* IC_50_ and *in vivo* dose highlights poor bioavailability. PK studies are urgently needed	Jiang et al. (2015)
	Chikusetsusaponin V	*In vitro*: HT29 cell migration assay Control: PBS vehicle	20–100 μg/mL (migration IC_50_ = 75 μg/mL)	24 h	↓ αvβ6 integrin expression; ↓ MMP-2/9 activity; suppression of ERK phosphorylation		Jiang et al. (2017)
Liver cancer	Deglucosyl chikusetsusaponin IVa	*In vitro*: HepG2 hepatocellular carcinoma cells Controls: Vehicle; positive (cisplatin, 12.7 μg/mL)	0.02–1.0 μmol/mL (IC_50_ = 0.06 μmol/mL)	24 h	↑ Bax expression; ↓ Bcl-2; G2/M cell cycle arrest; mitochondria-mediated apoptosis	High IC_50_ may limit clinical relevance. Heat treatment increases content and activity	Song et al. (2015)
	TSPJ	*In vivo*: H22 allograft mouse model Controls: Untreated; positive (5-FU, 20 mg/kg)	50–200 mg/kg/day (oral gavage)	14 days	↑ NK cell cytotoxicity; ↓ TGF-β, IL-10, and PGE2; tumor growth inhibition (62.3% at 200 mg/kg)		Shu et al. (2018)
Cervical cancer	Aqueous PJR extract	*In vitro*: HeLa cells Controls: Untreated; positive (paclitaxel, 1 μM)	100–500 μg/mL (IC_50_ = 200 μg/mL)	48 h	G0/G1 phase arrest; ↑ Bax/Caspase-3; ↓ Bcl-2; p53-dependent apoptosis	—	Chen (2018)
Ovarian cancer	Chikusetsusaponin IVa methyl ester	*In vitro*: A2780 cells Controls: Vehicle (DMSO); positive (paclitaxel, 10 nM)	4–20 μM (IC_50_ = 7.43 ± 1.55 μM)	24 h	↓ cyclin D1/CDK2/CDK6; ↑ cleaved caspase-3/PARP; ↓ MMP-2/9 enzymatic activity; inhibition of Cdc42/Rac/RhoA GTPases	—	Chen et al. (2016)
Prostate cancer	Chikusetsusaponin IVa	*In vitro*: PC-3 cells *In vivo*: PC-3 xenograft in athymic nude mice Control: Vehicle	*In vitro*: 12.5–50 μM *In vivo*: 15–60 mg/kg (oral)	24 h (cells) 7 weeks (mice)	↑ intracellular ROS; cytochrome c release; caspase-dependent/independent apoptosis; ↑ AIF/Endo G nuclear translocation; tumor suppression (58.2% at 60 mg/kg)	Selective toxicity to cancer cells is promising. PK profile unknown	Zhu et al. (2017)
Colorectal cancer	Chikusetsusaponin IVa methyl ester	*In vitro*: HCT116 cells Control: Wnt inhibitor (XAV939, 10 μM)	12.5–100 μM (β-catenin inhibition EC_50_ = 30 μM)	24 h	↓ Nuclear β-catenin translocation; ↓ cyclin D1/CDK2/CDK4; G0/G1 cell cycle arrest	—	Lee et al. (2015)
Gastric cancer	Chikusetsusaponins IV, IVa, V	*In vitro*: SGC-7901 cells Controls: Medium; positive (cisplatin, 5 μg/mL)	2.5–10 μM (chikusetsusaponin IVa IC_50_ = 5.2 μg/mL)	24 h	Dose-dependent anti-proliferation (IVa most potent); apoptosis induction (IV most effective)	—	Zhang et al. (2019)
Breast cancer	CSIVa-loaded liposome nanoparticles	*In vitro*: MDA-MB-231 cells *In vivo*: MDA-MB-231 xenografts Control: Empty liposomes	5–20 μM (IC_50_ = 7.5 μM)	48 h (cells) 21 days (mice)	↓ PI3K/Akt signaling; G2/M arrest; enhanced tumor penetration (73.6% tumor suppression *in vivo*)	Example of formulation improvement:Liposomal encapsulation enhances targeting and reduces toxicity, addressing PK limitations of free saponins	Xie et al. (2021)

### 4.1 Lung cancer

Lung cancer represents the most frequently diagnosed malignancy worldwide and constitutes the primary cause of cancer-related mortality among all tumor types (Mohindra and Patel, 2022). Recent pharmacological investigations have provided evidence of the potent anti-Lung cancer activities exhibited by TSPJ (Zhu et al., 2024). *In vitro* studies show that TSPJ exerts antiproliferative and proapoptotic effects on human lung adenocarcinoma A549 cells. These effects are mediated by concentration-dependent upregulation of Caspase-3, a critical protease involved in apoptosis (Hong-Yan et al., 2015). Additionally, TSPJ demonstrates antitumor activity in rat models of Lung cancer. Its mechanisms involve regulation of the TLR4/NF-κB pathway, reduction of inflammatory cytokines, and enhancement of immune function (Gao et al., 2020a). While Jiang et al. (2015) linked TLR4/NF-κB inhibition to TSPJ’s effects, their study lacked genetic validation (e.g., TLR4 knockout controls). Dose discrepancies between in vitro (IC_50_ = 50 μg/mL) and in vivo (200 mg/kg) regimens suggest limited bioavailability—unaddressed in current Pharmacokinetic studies. Furthermore, Gao et al. (2020a) reported PKCα-ERK1/2 inhibition without testing pathway crosstalk with PI3K/Akt, potentially oversimplifying the mechanism.

However, a marked dose discrepancy exists between the *in vitro* half-maximal inhibitory concentration *(IC*_*50*_ *= 50 μg/mL)* of TSPJ in A549 cells and the *in vivo* administration dose (200 mg/kg), highlighting a potential limitation in translational relevance. This discrepancy likely stems from the complex pharmacokinetic processes in living organisms, including oral bioavailability, tissue distribution, and first-pass metabolism. The current experimental design has not directly validated the achievable concentrations of the *in vitro* effective dose *in vivo*, which constitutes a significant limitation. Future investigations should incorporate pharmacokinetic analyses to establish a more precise dose-response relationship between *in vitro* and *in vivo* conditions.

Further studies have indicated that TSPJ prominently suppresses the proliferation of human lung adenocarcinoma A549 cells through mechanisms involving the PI3K/Akt pathway, particularly by modulating PTEN, a known tumor-suppressing factor. The PI3K/Akt signaling cascade, a critical intracellular pathway, controls diverse cellular processes. TSPJ treatment increases the expression of the PTEN gene, consequently reducing PI3K phosphorylation, thereby diminishing Akt activation. Reduced Akt activity subsequently interferes with downstream signals crucial for tumor cell growth and proliferation. Additionally, TSPJ has shown efficacy in attenuating cancer cell migration and invasion through downregulation of MMP-2 and MMP-9, two matrix-degrading proteases. By limiting these enzymes’ activities, TSPJ effectively prevents degradation of extracellular matrix (ECM) metabolites, thereby restricting tumor metastasis and invasion (Gao et al., 2020b).

**FIGURE 5:**
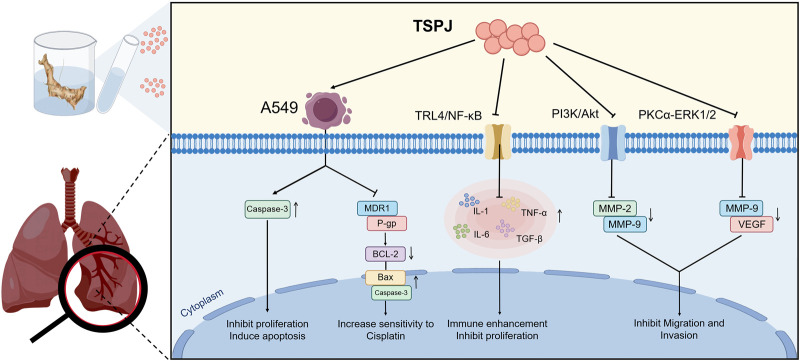
Multitarget mechanisms of TSPJ against Lung cancer. TSPJ upregulates Caspase-3 in A549 cells, inhibits proliferation, and induces apoptosis. Meanwhile, TSPJ inhibits MDR1 and P-gp, downregulates Bcl-2, upregulates Bax and Caspase-3, and enhances cellular sensitivity to cisplatin. In addition, TSPJ regulates the TLR4/NF-κB pathway to reduce IL-1, IL-6, TNF-α, and TGF-β levels, improving immune function. It also reduces MMP-2, MMP-9, and VEGF through the PI3K/Akt and PKCα-ERK1/2 pathways, thus suppressing tumor growth and invasion.

Moreover, research suggests TSPJ suppresses Lewis Lung cancer cell growth and invasive potential by inhibiting the PKCα-ERK1/2 pathway, accompanied by reduced VEGF and MMP-9 expression (Gao et al., 2020c). Significantly, TSPJ also considerably enhances Lung cancer cells’ chemosensitivity. For instance, in cisplatin-resistant A549DDPR cells, TSPJ administration improves their responsiveness to cisplatin by reducing expression of the multidrug resistance gene 1 (MDR1) and P-glycoprotein (P-gp), increasing pro-apoptotic proteins Caspase-3 and Bax, and suppressing anti-apoptotic protein Bcl-2 (Gao et al., 2020d) (Figure 5).

Although existing studies have preliminarily elucidated that Zhujieshen exerts anti-tumor effects through PI3K–Akt, PKCα–ERK1/2, and TLR4/NF-κB pathways, these pathways do not operate in isolation but constitute a dynamically interconnected regulatory network. For instance, both PI3K–Akt and ERK1/2 pathways are activated upstream by epidermal growth factor receptor (EGFR) and other growth factor receptors, while Akt can indirectly inhibit ERK1/2 activity by phosphorylating RAF, forming a negative feedback loop (Dhillon et al., 2007). Conversely, activation of NF-κB is positively regulated by PKCα (Roy et al., 2013), and Akt can promote NF-κB nuclear translocation by phosphorylating IκB kinase (IKK) (Tanaka et al., 2005), thereby amplifying the secretion of pro-inflammatory cytokines such as TNF-α and IL-6, which further activates the PI3K–Akt pathway to form a positive feedback loop (Song et al., 2017). Monomeric components of Zhujieshen, such as TSPJ and CSIV, may disrupt these pro-tumor networks by simultaneously inhibiting multiple nodes. For example, TSPJ not only downregulates PI3K–Akt but also inhibits PKCα–ERK1/2, synergistically blocking cell cycle progression. Its inhibition of TLR4/NF-κB may indirectly reduce Akt activation sources, enhancing chemosensitivity (Figure 5). However, current research still lacks direct evidence to validate the cross-regulatory relationships of these pathways under Zhujieshen intervention, particularly due to the absence of *in vivo* genetic experiments, leaving network-level mechanistic inferences at the hypothetical stage. Future studies should employ multi-omics integrative analysis and gene-editing techniques to further elucidate its systems pharmacology mechanisms.

### 4.2 Liver cancer

Among all cancer types globally, liver cancer occupies the seventh position; however, in terms of cancer-associated mortality, it ranks second, preceded only by Lung cancer (McGlynn et al., 2021; Ferlay et al., 2015). Importantly, approximately 45%–50% of the global liver cancer diagnoses arise in China (Jin et al., 2022; Sung et al., 2021). Bioactive derivatives and total saponins extracted from Panacis Japonici Rhizoma (TSPJ) display significant antitumor effects, which include triggering apoptosis, suppressing cellular proliferation, and hindering metastatic capabilities of liver cancer cells. Research employing mouse models with transplanted H22 hepatic tumors showed pronounced tumor growth inhibition and notably extended lifespan following administration of TSPJ (Deng et al., 2013; Yuan et al., 2007). Furthermore, a study indicated that Zhujieshen-containing serum exerts pronounced effects on human hepatocellular carcinoma cell lines, resulting in substantial antiproliferative activity and cell-cycle arrest at the G0/G1 checkpoint (Huang, 2017).

Increasing evidence emphasizes the anticancer efficacy of bioactive CSIV and CSV metabolites from Zhujieshen against hepatic carcinoma. Experimental *in vitro* evidence demonstrated a clear dose-related inhibition of HepG2 cellular proliferation mediated by these saponins. The underlying mechanisms include disruption of mitochondrial membrane potential, induction of apoptosis, alteration in intracellular calcium levels, modulation of apoptosis-associated proteins, and blockage of the cell cycle. Specifically, CSV and CSIV induce apoptosis predominantly via p53-dependent pathways, upregulating pro-apoptotic markers including cytochrome c (Cyt c), p21, p53, cleaved caspase-3/-9, and Bax, while concurrently diminishing anti-apoptotic Bcl-2 protein levels (Zuo et al., 2022). Another independent study demonstrated that CSIVa effectively inhibits proliferation of SK-Hep-1 hepatocellular carcinoma cells, with an IC_50_ value of 18.9 μg/mL (Yoo et al., 2006). Additional experiments indicated that deglucose CSIVa (DCIVa) extracted from Zhujieshen markedly reduced HepG2 cell viability in both concentration- and time-dependent manners. Furthermore, Hoechst 33258 staining revealed distinct apoptotic features in DCIVa-treated cells, such as nuclear condensation, chromatin marginalization, and apoptotic body formation. DCIVa facilitates apoptosis through elevating pro-apoptotic Bax and diminishing anti-apoptotic Bcl-2 protein expression. Additionally, DCIVa induces cell-cycle arrest at the G2/M checkpoint, thereby effectively suppressing tumor cell proliferation (Song et al., 2015). While Huang, (2017) demonstrated cell cycle arrest using aqueous extracts, the absence of saponin profiling precludes attributing the observed effects to specific metabolites. Conversely, Song et al. (2015) employed rigorously HPLC-validated deglucosyl CSIVa; however, the reported inhibitory concentration (IC_50_ = 0.06 μmol/mL) exceeds physiologically relevant drug levels. This necessitates dose-escalation studies to establish translational relevance.

Song et al. (2015) reported that the half-maximal inhibitory concentration (IC_50_ = 0.06 μmol/mL) of DCIVa against HepG2 cells was relatively high. However, whether this concentration corresponds to clinically achievable and safe plasma levels remains unknown. Therefore, while the study provides valuable mechanistic insights, the physiological and clinical relevance of its findings requires confirmation through subsequent dose-exploration and pharmacokinetic studies.

Further studies suggest that Zhujieshen extracts enhance immune recognition and cytotoxic activity against tumor cells. Specifically, aqueous Zhujieshen extracts significantly improve the thymus and spleen indices in mouse liver cancer models, boosting systemic immunity (Huang, 2017). Subsequent research on TSPJ revealed concentration-dependent enhancement of natural killer (NK) cell and CD8^+^ T-cell sensitivity toward H22 mouse hepatoma cells, improvement of CD4^+^ T-cell apoptosis, normalization of cytokine secretion profiles, and reduced immunosuppressive factors, thus strengthening immune responses (Shu et al., 2018; Chen et al., 2014).

**FIGURE 6:**
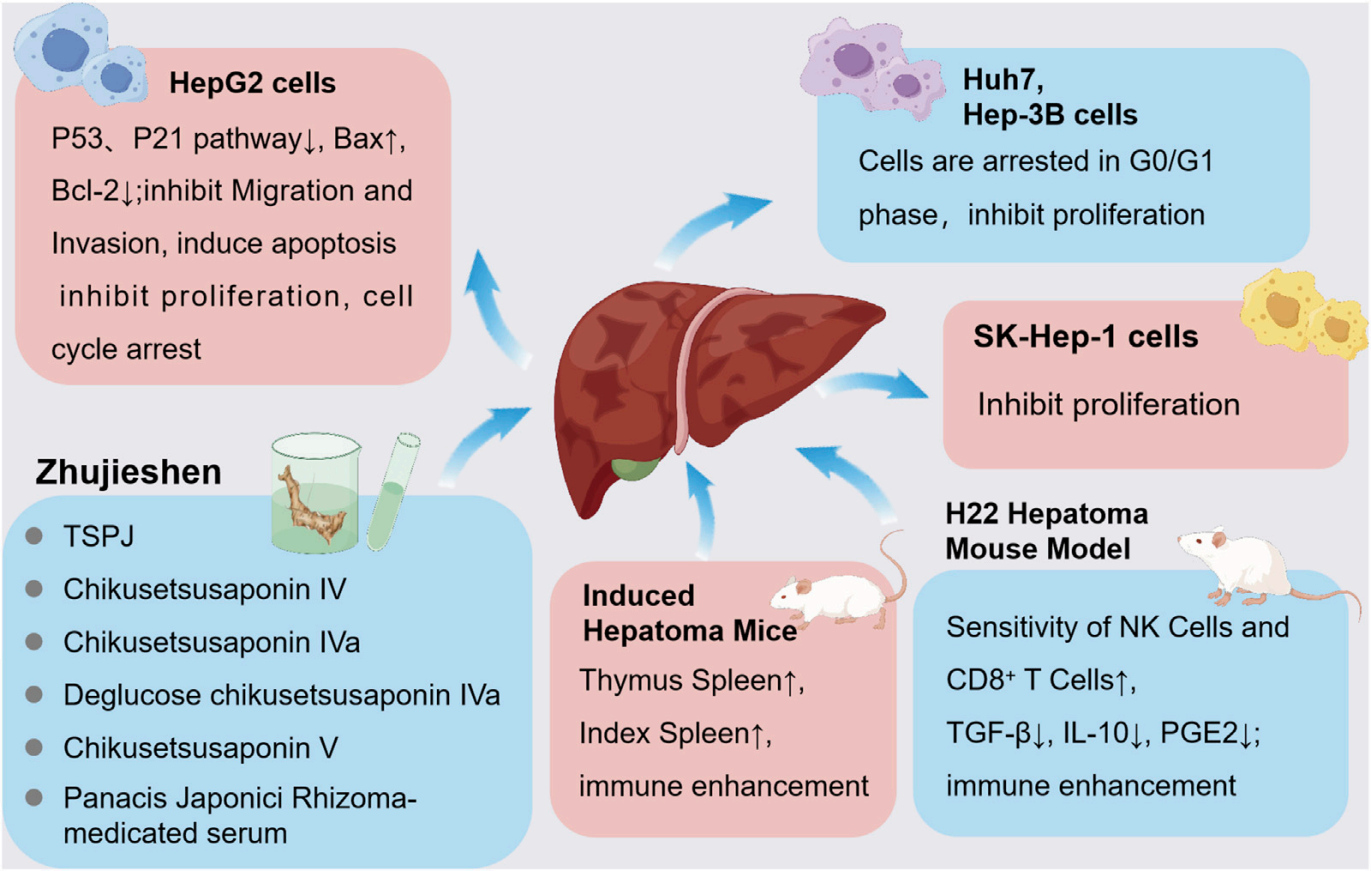
Anti-liver cancer activity. Zhujieshen induces G0/G1-phase arrest, regulates p53 and p21 signaling, modulates Bax and Bcl-2 expression to inhibit cell migration and invasion, induces apoptosis, and suppresses cell proliferation. Additionally, Zhujieshen enhances the thymus and spleen indices, improves NK and CD8^+^ T-cell sensitivity, and reduces immunosuppressive cytokines TGF-β, IL-10, and PGE2, thus strengthening immune function.

Network pharmacology studies have hypothesized that Zhujieshen may prevent and treat liver cancer by regulating critical molecules such as Akt1 and Caspase-3. However, this hypothesis requires further experimental validation (Song et al., 2023). Another study indicated that metabolites from thermally processed Zhujieshen exhibit stronger inhibitory effects on DEN-induced hepatoma cells compared to ginseng at equivalent concentrations (Kim et al., 2013). Research by Yoo et al. further supports this, indicating that heat treatment significantly increases CSIVa content in Zhujieshen extracts, markedly enhancing cytotoxic activity (Yoo et al., 2006) (Figure 6).

Zhujieshen has attracted considerable attention due to its abundant bioactive metabolites. However, current experimental studies on liver cancer involve diverse metabolites without standardized quality evaluation criteria. To precisely assess Zhujieshen’s potential for liver cancer treatment, future research should focus on characterizing specific active fractions or isolated bioactive metabolites. This approach would provide clearer clinical guidelines and a robust theoretical basis for subsequent studies.

### 4.3 Cervical cancer (CC)

CC is a common malignancy among women, ranking fourth globally in cancer-related mortality (Filho et al., 2023) and first among reproductive system cancers (Jiang et al., 2024). Studies have demonstrated that aqueous extracts of Zhujieshen significantly inhibit proliferation of human CC HeLa cells (Ni, 2007). Moreover, recent findings indicated that aqueous Zhujieshen extracts decrease HeLa cell proliferation rates and induce early-stage apoptosis in a concentration-dependent fashion. Proposed underlying mechanisms involve: (1) Induction of cell-cycle arrest: cells treated with aqueous Zhujieshen extracts accumulate predominantly in the G0/G1 phase, with a concomitant reduction of cells progressing into the S phase, thus suppressing proliferation (Chen Y. et al., 2023); (2) Regulation of apoptosis-associated proteins: the extracts stimulate expression of pro-apoptotic Bax, suppress anti-apoptotic Bcl-2, and enhance Caspase-3 activation, thereby facilitating apoptotic processes (Chen, 2018; Chen et al., 2016); and (3) Alteration of gene expression profiles: extracts significantly elevate mRNA levels of apoptosis-related genes (P53, Bax, Caspase-3), promoting both autophagic and apoptotic cell death pathways (Liu Y. et al., 2016). All cited studies base for these mechanisms relies exclusively on *in vitro* studies (Chen, 2018; Chen et al., 2016) utilizing HeLa cells, which harbor aberrant p53 signaling—a critical caveat limiting extrapolation. The applied doses (100–500 μg/mL) lack physiological relevance, and reported apoptosis markers (Bax/Bcl-2) were not corroborated by functional endpoints (Huang, 2021).

In summary, Zhujieshen demonstrates substantial potential against CC by inhibiting cell proliferation and inducing apoptosis. However, current investigations remain limited to *in vitro* models, lacking preclinical animal studies and clinical evidence. This gap significantly restricts translational relevance, as *in vitro* models inadequately represent human physiological complexity, pharmacokinetics, and toxicity. Consequently, existing evidence is preliminary and insufficient for clinical application. Future research must prioritize investigating the anticancer effects of Zhujieshen’s active fractions or isolated metabolites through *in vivo* models and clinical trials. Such studies will validate these mechanisms and provide reliable foundations for therapeutic strategies.

### 4.4 Ovarian cancer (OC)

OC, a common malignancy in women, poses a significant threat to women’s health due to its high mortality rate (Prat, 2012). Studies indicate that TSPJ inhibits the proliferation of OC HEY cells (Liu J. L. et al., 2016). Additionally, related research highlight the antitumor potential of oleanolic acid derivatives and CSIVa methyl ester (CSME), both isolated from Zhujieshen, in OC treatment. Specific details are described below:

1. CSV butyl ester exhibits moderate antitumor efficacy towards OC cells OVCAR-3 and A2780, showing respective IC_50_ values of 35.2 and 21.1 μg/mL. Despite its observed anticancer potential, the detailed underlying mechanisms still warrant additional clarification (Zhao et al., 2010);
2. CSME presents notable cytotoxic effects on OC cell lines HEY and A2780, with IC_50_ values consistently below 10 μmol/L. The antiproliferative action of CSME involves induction of G1-phase arrest, significantly reducing the population of cells in DNA synthesis (S-phase). Treatment with CSME concurrently decreases mitochondrial membrane integrity, elevates Annexin V-positive apoptotic cell proportions, and results in nuclear chromatin condensation—characteristic morphological features associated with apoptosis. Further mechanistic studies have shown that CSME elevates apoptotic proteins such as activated Caspase-3, Bax, and cleaved PARP, while concurrently suppressing anti-apoptotic Bcl-2 protein levels, thus facilitating programmed cell death. Additionally, OC cell proliferation, invasion, and migration are markedly impeded by CSME via downregulation of cell-cycle regulating proteins and attenuation of MMP2 and MMP9 enzymatic activities (Chen et al., 2016).

### 4.5 Prostate cancer (PCa)

PCa represents a commonly occurring malignancy within the male reproductive and urinary systems (Wang et al., 2022). CSIVa, one of the primary bioactive molecules isolated from Zhujieshen, demonstrates significant antiproliferative capacity against PCa cells while inducing apoptosis and exhibiting limited toxic effects on healthy prostate epithelial cells (Zhu et al., 2017). Treatment with CSIVa results in enhanced intracellular ROS accumulation in PCa cells (Kim et al., 2016). The excessive generation of ROS disrupts mitochondrial membrane stability, provoking mitochondrial expansion and rupture, followed by release of apoptotic effectors and subsequent apoptosis initiation.

**FIGURE 7:**
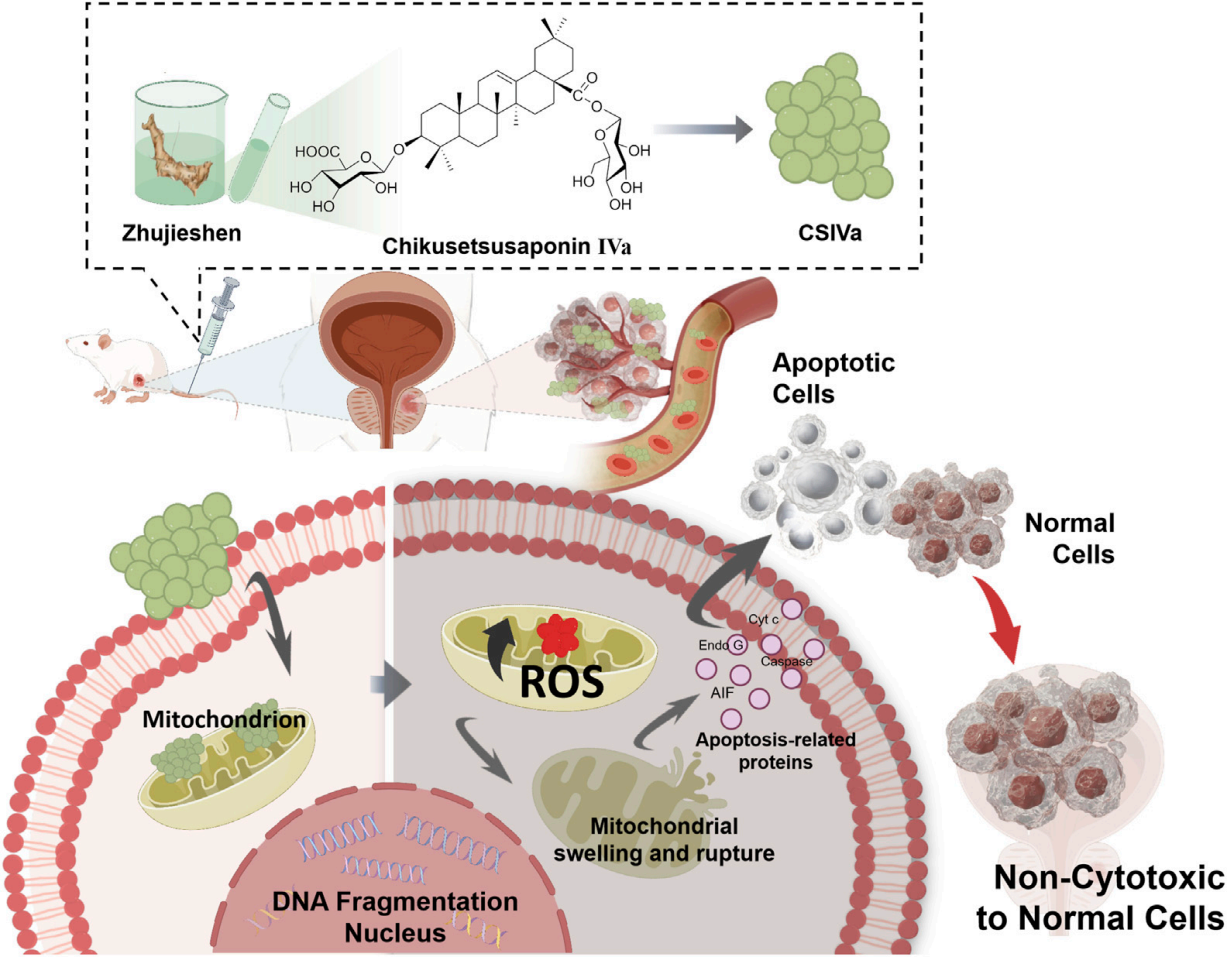
Anti-PCa activity. CSIVa selectively inhibits PCa cell proliferation and induces apoptosis without harming normal cells. CSIVa treatment in PCa cells triggers ROS production, mitochondrial dysfunction, Cyt c release, and subsequent induction of apoptosis via both caspase-dependent and caspase-independent pathways. Additionally, CSIVa promotes nuclear translocation of AIF and Endo G, causing DNA fragmentation and ensuring complete cell death.

Both caspase-independent and caspase-dependent pathways mediate CSIVa-induced apoptosis. In the caspase-dependent process, CSIVa stimulates Cyt c release from mitochondria into the cytoplasm, where Cyt c interacts with Apaf-1, forming an apoptosome that activates caspase cascades to cleave and stimulate apoptotic proteins. Additionally, CSIVa enhances caspase expression, further amplifying apoptosis. Conversely, the caspase-independent apoptotic route involves CSIVa-induced nuclear translocation of apoptosis-inducing factor (AIF) and endonuclease G (Endo G). Both molecules play crucial roles in late-stage apoptosis, causing nuclear DNA fragmentation and ensuring complete cell death (Zhu et al., 2017) (Figure 7).

### 4.6 Colorectal cancer (CRC)

CRC, a prevalent gastrointestinal malignancy, ranks third in global incidence, following lung and BC (Arnold et al., 2017; Bray et al., 2018). To investigate the potential of TSPJ against cancer-associated cachexia, Zhou and colleagues established a mouse model by subcutaneous inoculation of CT26 colon adenocarcinoma cells. Cachexia, a frequent syndrome in advanced cancer, involves significant weight loss and rapid depletion of muscle and adipose tissue. The results indicated that TSPJ significantly increased body weight, prevented muscle and fat degradation, and improved cachectic symptoms. Additionally, TSPJ inhibited the NF-κB-mediated inflammatory response by downregulating TNF-α and IL-1 (Zhou et al., 2018).

CSV, a bioactive saponin isolated from Zhujieshen, suppresses HT29 colon cancer cell metastasis to the lungs by reducing cell migration, invasion, and adhesion capabilities. Its antimetastatic effects are mediated through the inhibition of integrin αvβ6, matrix metalloproteinases MMP-2 and MMP-9, and phosphorylation of ERK, with minimal cytotoxic effects (Wang et al., 2020; Jiang et al., 2017). Another active metabolite, CSME, acts as a novel suppressor of colon cancer HCT116 cell growth by targeting the Wnt/β-catenin pathway. Specifically, CSME decreases nuclear β-catenin concentrations and inhibits its interaction with the TCF binding elements (TBE) of target gene promoters. Given β-catenin’s crucial function in cellular proliferation, CSME reduces Cyclin D1, CDK2, and CDK4 expression, thus arresting cell cycle progression at the G0/G1 phase and consequently inhibiting proliferation (Lee et al., 2015).

### 4.7 Other tumors

Zhujieshen demonstrates potential pharmacological effects in renal cell carcinoma, gastric cancer, BC, neuroblastoma, and leukemia (Ru et al., 2022; Wang et al., 2012). 1. TSPJ markedly suppresses renal cancer ACHN and A498 cell proliferation, migration, and invasion, while also inducing apoptosis. The underlying mechanism is attributed to decreased expression of angiotensin II (Ang II) and its receptor (AT1R), subsequently affecting downstream VEGF and COX-2 pathways (Zhang et al., 2019); 2. Gastric cancer: Triterpenoid saponins isolated from Zhujieshen exhibit dose-dependent suppression of proliferation, migration, and invasive capabilities in gastric cancer SGC-7901 cells, simultaneously promoting apoptosis. Among these metabolites, CSIVa demonstrates the strongest antiproliferative efficacy, whereas CSIV shows more pronounced apoptotic induction than other saponins evaluated (Xie et al., 2021; Zhang et al., 2019); 3. BC: Liposome nanoparticles containing CSIVa combined with the photosensitizer chlorin e6 markedly inhibit BC cell growth and trigger apoptosis, demonstrating minimal toxic effects towards healthy cells (Monaco et al., 2023). In triple-negative BC cell line MDA-MB-231, CSIVa mediates suppression of proliferation, induction of apoptosis, cell-cycle arrest at the G2/M checkpoint, and downregulation of the PI3K/Akt signaling cascade, without notable cytotoxicity to normal breast epithelial cell line MCF-10A (Liang et al., 2023); 4. Human neuroblastoma: CSV exhibits neuroprotective and antioxidant capacities through modulation of the SIRT1/PGC-1α/MnSOD pathway, alleviating oxidative injury induced by H_2_O_2_ in SH-SY5Y cells (Wan et al., 2016); 5. Leukemia: Yuan et al. demonstrated that TSPJ inhibits proliferation of leukemia HL-60 cells, inducing G0/G1-phase arrest and differentiation into granulocytes (Ding and Zhang, 2007).

## 5 Safety, toxicity, and dosing considerations

Notwithstanding the promising antitumor activities outlined above, the translational potential of Zhujieshen is inextricably linked to its safety profile and bioavailability—aspects that current research has yet to sufficiently address. Isolated findings hint at a favorable therapeutic window; for instance, CSIVa’s selective cytotoxicity against cancer cells (e.g., PC-3, MDA-MB-231) with minimal impact on corresponding normal epithelial cells provides a preliminary safety signal (Zhu et al., 2017; Wan et al., 2016). Furthermore, the reported efficacy of TSPJ in the H22 allograft model at an oral dose of 200 mg/kg/day over 14 days, without mention of overt toxicity, suggests a tolerable dose range in mice (Shu et al., 2018). Nevertheless, these observations are incidental. A systematic toxicological evaluation—defining the maximum tolerated dose (MTD), and assessing chronic toxicity and organ-specific impacts (particularly hepatorenal function)—remains a critical gap. The considerable disparity between *in vitro* IC_50_ values (often in the μg/mL range) and the required *in vivo* doses underscores the inherently low bioavailability of its saponin constituents. Therefore, future work must prioritize rigorous pharmacokinetic/pharmacodynamic (PK/PD) studies to bridge this gap and inform viable clinical dosing strategies.

## 6 Conclusion and future perspectives

Preclinical studies underscore the multitarget antitumor potential of Zhujieshen and its bioactive saponins, including TSPJ, CSIV, and CSIVa. These compounds exhibit activity across diverse malignancies by modulating critical oncogenic processes—such as proliferation, apoptosis, metastasis, and chemoresistance—primarily through interactions with key signaling pathways, including PI3K/Akt, PKCα-ERK1/2, and TLR4/NF-κB.

However, clinical translation remains hindered by significant pharmacokinetic (PK) challenges, including poor solubility, low oral bioavailability, and rapid clearance, which explain the stark discrepancies between *in vitro* effective concentrations and *in vivo* exposure levels. The pharmacological relevance of *in vitro* “effective concentrations” necessitates rigorous validation via *in vivo* PK/PD models. Without such corroboration, extrapolating experimental findings to complex biological systems remains speculative, particularly when considering multi-organ metabolic interactions and nonlinear PK behaviors. Furthermore, the synergistic mechanisms underlying Zhujieshen’s multicomponent composition are underexplored, and evidence for efficacy against certain cancers, such as CC, is currently limited to *in vitro* models, highlighting the need for robust preclinical validation.

To advance translational research, several strategic priorities emerge. First, standardized analytical protocols—such as LC-MS/MS—should be established for precise quantification of key saponins across botanical sources and extracts, ensuring batch consistency and reproducible pharmacological effects. Second, innovative formulation strategies, including liposomal or PEGylated nanoparticle systems, may enhance the solubility, stability, and tumor-targeted delivery of these hydrophobic saponins.

Promisingly, preliminary studies already point to viable solutions. Thermal processing of Zhujieshen has been shown to increase the content and cytotoxic activity of key saponins like CSIVa, suggesting a simple method to enhance potency (Yoo et al., 2006). More advanced nano-encapsulation strategies, such as CSIVa-loaded liposomes tested in breast cancer models, demonstrate the potential to improve tumor targeting, enhance bioavailability, and reduce systemic toxicity by protecting the cargo and facilitating its accumulation at the tumor site (Xie et al., 2021). These formulation improvements are not futuristic concepts but necessary steps to bridge the gap between preclinical efficacy and clinical application.

Mechanistically, the multitarget nature of Zhujieshen aligns with systems pharmacology principles. Future studies should integrate network pharmacology and multi-omics approaches to empirically validate synergistic interactions among metabolites and map their collective impact on signaling networks driving tumorigenesis. Addressing PK/PD gaps requires standardized modeling frameworks to bridge preclinical-to-clinical translation, enabling dose optimization and biomarker identification.

Ultimately, these foundational efforts must culminate in well-designed clinical trials evaluating Zhujieshen-based therapies as monotherapy or in combination with standard regimens. Biomarker-driven patient stratification should be incorporated to objectively assess therapeutic efficacy and quality-of-life outcomes. By systematically addressing standardization, formulation, mechanistic elucidation, and clinical validation, the potential of Zhujieshen as a multifaceted anticancer agent may be rigorously evaluated for integration into modern oncology practice.

## Glossary

AIF: Apoptosis-Inducing Factors
Akt: Protein kinase B
Ang II: Angiotensin II
Apaf-1: Apoptotic protease activating Factor-1
AT1R: Angiotensin II type 1 receptor
Bax: BCL2-associated X protein
Bcl-2: B-cell lymphoma-2-related protein
Caspase-3: Cysteine-requiring aspartate Protease-3
CCK-8: Cell counting Kit-8
Cdc42: Cell division cycle 42
CDK2: Cyclin dependent kinase 2
CDK4: Cyclin dependent kinase 4
CDK6: Cyclin dependent kinase 6
CSIVa: Chikusetsusaponin IVa
CSIV: Chikusetsusaponin IV
CSV: Chikusetsusaponin V
Cyt c: Cytochrome C
CSME: Chikusetsusaponin IVa methyl ester
Endo G: Endonuclease G
ERK: Extracellular signal-regulated kinase
GSK-3β: Glycogen synthase Kinase-3β
ICAM-1: Intercellular adhesion Molecule-1
IL-1: Interleukin-1
IL-6: Interleukin-6
IL-10: Interleukin-10
MAPK: Mitogen-activated protein kinase
MMP-2: Matrix Metalloproteinase-2
MMP-9: Matrix Metalloproteinase-9
MDR-1: Multidrug Resistance gene 1
MMP: Matrix metalloproteinase
NF-κB: Nuclear Factor-κB
NK: Natural killer cells
PARP: Poly (ADP-ribose) Polymerase
PGE2: Prostaglandin E2
PI3K: Phosphoinositide 3-kinase
PTEN: Phosphatase and tensin homolog
ROS: Reactive oxygen species
TGF-β: Transforming growth factor-β
TNF-α: Tumor necrosis factor-α
TSPJ: Total saponins of Panacis japonici rhizoma
TCF: T Cell factor
VEGF: Vascular endothelial growth factor
